# A Comparative Study of Two-Minute versus Three-Minute Passive Recovery on Sprint Skating Performance of Ice Hockey Forwards and Defensemen

**DOI:** 10.3390/ijerph182413029

**Published:** 2021-12-10

**Authors:** Arkadiusz Stanula, Subir Gupta, Jakub Baron, Anna Bieniec, Rajmund Tomik, Tomasz Gabrys, Petr Valach, Andrzej Szymon Swinarew

**Affiliations:** 1 Institute of Sport Sciences, Jerzy Kukuczka Academy of Physical Education, 40-065 Katowice, Poland; j.baron@awf.katowice.pl (J.B.); a.bieniec@awf.katowice.pl (A.B.); r.tomik@awf.katowice.pl (R.T.); 2 Faculty of Medical Sciences, Cave Hill Campus, University of West Indies, Bridgetown BB11000, Barbados; subir.gupta@cavehill.uwi.edu; 3 Department of Physical Education and Sport, Faculty of Education, University of West Bohemia, 30100 Pilsen, Czech Republic; tomaszek1960@o2.pl (T.G.); pvalach@ktv.zcu.cz (P.V.); 4 Faculty of Science and Technology, University of Silesia in Katowice, 41-500 Chorzów, Poland; andrzej.swinarew@us.edu.pl

**Keywords:** heart rate, field test, skating performance, movement pattern, performance decrement, fatigue index

## Abstract

The impact of two different passive recovery durations, two and three minutes, between sets of repeated sprint skating ability (RSSA) test on skating speed, speed decrement (S_dec_), and heart rate (HR) response of ice hockey forwards (n = 12) and defensemen (n = 7) were determined. Six sets of 3 × 80 m sprint, with two-minute passive recovery between two consecutive sets, were performed in RSSA-2. A three-minute passive recovery period between two consecutive sets was allowed in RSSA-3. Skating speed, S_dec_, and HR were measured in all tests. Subjects skated faster (*p* < 0.05) in most of the RSSA-3 sets than the corresponding set of RSSA-2. Defensemen were slower (*p* < 0.05) than forwards in most of the cases. The S_dec_ was higher in defensemen than in forwards, although the difference was significant occasionally. No difference in peak HR and minimum HR between forwards and defensemen was found. RSSA-3 is beneficial over RSSA-2 in both forwards and defensemen by increasing speed. Defensemen are slower and show early fatigability than forwards, and no difference in HR response was noted between forwards and defensemen. This study concludes that three-minute recovery is beneficial over two-minute recovery by increasing skating speed, although S_dec_ and HR response neither vary significantly between RSSA-2 and RSSA-3, nor between forwards and defensemen.

## 1. Introduction

Repeated sprint skating, quick changes in direction, and frequent profound body contact are some major characteristics of the game of ice hockey. A standard ice hockey match is actually played for 60 min, which is made up of three 20 min periods. The total time of play, however, often extends much longer, including two rest intervals [1,2,3]. To keep the game fast, frequent substitution of players is a common practice in ice hockey matches. On average, an ice hockey player effectively plays for 15 to 24 min, which is relatively shorter than players of most other team sports of comparable duration [4]. In ice hockey matches, the players do not play continuously but alternate at nearly regular intervals, commonly known as a ”shift”. The duration of each shift usually varies from 30 to 80 s, interspersed with 2 to 5 min of passive recovery [4].

Ice hockey players of various playing positions experience different workloads that can reflect their physiological profiles. Researchers conducted a number of studies on physical- and physiological profiles of ice hockey players in relation to their position of play [5,6,7]. Researchers reported similar aerobic capacity in ice hockey forwards and defensemen, but lower values in goalkeepers [5,8,9,10]. Similarly, difference in body composition and anthropometric parameters were also reported on ice hockey players of different playing positions [5].

Sport scientists and trainers have designed a number of repeated sprint ability (RSA) tests based on the movement patterns and speed at the intense period of competitive match play in team sports [11,12,13,14,15]. Despite an exponential increased interest, the physiological basis of drop-off performance in RSA test remains obscured [16]. Repeated sprint performance, however, is useful in guiding and implementing appropriate training that likely improves match performance by deferring fatigue [17].

Several on-ice RSA tests, on a wide range of ice hockey players, have been introduced by sports scientists and strength and conditioning coaches. Repeated sprint skating ability (RSSA) test was designed in this study to replicate some of the basic movement patterns and recovery period during ice hockey match play. Duration of recovery period or bench time, between shifts of play, are expected to play an important role in the extent of recovery and thus skating performance during the shift of play. Replacement of the players, or rest period between shifts, are strategic and aimed to allow the players to recover sprint ability [9]. However, to the best of our knowledge, no such studies were carried out on the ice hockey players that show the effect of different recovery period duration on players of different positions in real match play or any form of RSSA tests.

This study is designed to mimic some basic movement patterns and passive recovery period of ice hockey game with RSSA protocol. The major aim of this study is to determine the effect of two different durations of recovery periods, 2 min (or RSSA-2) and 3 min (or RSSA-3), between successive sets of repeated sprint skating ability test. The impact of the recovery period duration on the skating performance of ice hockey forwards and defensemen was studied by analyzing average skating speed, speed decrement (S_dec_), and heart rate during RSSA-2 and RSSA-3 tests.

## 2. Materials and Methods

### 2.1. Subjects

All the subjects of this study were selected from the club Zagłębie Sosnowiec, participating in the highest ice hockey league in Poland. Uninjured outfield players (12 forwards, 7 defensemen), having a minimum experience of 7 years of playing ice hockey, and those who participated at least 90% of the training sessions, were finally selected as the subjects for this study. Players were training in a regular scheme of off-season macrocycle, which involved 2–3 gym sessions per week and lasted about 60 min, with average RPE around 7 (in 1–10 scale). They had also one functional—prehab training, lasting around 30 min with average RPE of 3.

### 2.2. Main Test Procedures

#### 2.2.1. Study Design

This study was conducted in the preseason of 2020, over 4 nonconsecutive days. On day 1, height, body weight, and body composition of the subjects were measured. Skating Multistage Aerobic Test (SMAT) was used to determine maximum heart rate (HR_max_) and predict VO_2max_ on day 2. On days 3 and 4 (separated by 5 days), repeated sprint skating ability (RSSA), by using two different durations of passive recovery period (2 and 3 min) between sets of RSSA were measured. Experiments on days 2, 3, and 4 were conducted at the same time of the day (10 a.m. to 1 p.m. and 6 p.m. to 9 p.m.). Subjects were informed to abstain from training and any other forms of heavy physical exercise for 24 h before the experiment. They were also instructed to maintain normal diet and fluid intake. The protocol and possible risks involved in the study were explained to the subjects before they gave voluntary written informed consent. The study was approved by the Ethics Committee of the Jerzy Kukuczka Academy of Physical Education in Katowice (approval number: 8/2018).

#### 2.2.2. Measurement of Body Composition

Body composition analyzer (InBody170, Biospace, Seoul, Korea) was used to determine body mass and height, total muscle mass, fat mass, and total body water of the subjects. All the subjects were tested at least 4 h after a meal, in empty bladder condition and at normal room temperature (20–23 °C).

#### 2.2.3. Measurement of Maximum Heart Rate and Predicted VO_2max_ by Skating Multistage Aerobic Test (SMAT)

Leone et al. [18] originally designed this on-ice test. The test was conducted on an indoor ice hockey rink, defined with markers at both extremities. The subject skated from one end to the other holding the hockey stick with the preferred one hand. The speed was increased gradually till he was unable to maintain it. The pace of the subject was dictated by audible signals emitted from a calibrated audio player. The starting speed was 3.5 m∙s^−1^, followed by stepwise increment by 0.2 m∙s^−1^. A passive recovery period of 30 s was allowed before the next sprint was started. The following formula was used to predict VO_2max_ of the subject [18]:

VO_2max_ = 18.07 × (maximum velocity in m∙s^−1^) − 35.596 mL∙kg^−1^∙min^−1^ (1)

#### 2.2.4. Repeated Sprint Skating Ability (RSSA) Test

**Figure 1:**
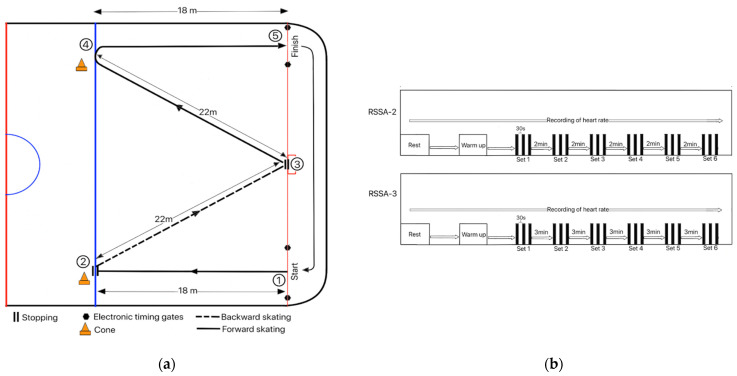
(**a**) Direction and distance of sprint skating in the repeated sprint skating ability test; (**b**) Schematic presentation of the overall study protocol. It illustrates 6 sets of RSSA tests with two different recovery periods between the sets—RSSA-2, with recovery period of 2 min, and RSSA-3, with recovery period of 3 min. It also shows that heart rate was recorded continuously, starting from rest till the end of the 6th set of both RSSA-2 and RSSA-3 tests.

Based on the movement pattern and duration of each sprint of the players found during ice hockey match play, Hůlka et al. [14] originally designed this test. Subjects were familiar with this test. They were required to perform 6 sets of sprint trials. Each set of sprint trial was composed of a 3 × 80 m sprint. The direction of skating of each repetition of sprint is shown in Figure 1a. Each repetition of sprint consisted of 18 m of skating forward straight from the goal line, stopping at blue line (2 m from barrier), followed by skating backward 22 m on the goal line, then skating forward 22 m up to blue line, followed by sharp right turn towards the goal line and the last 18 m skating forward to finish at the goal line. Thus, the total distance covered in each repetition of sprint was 80 m. After each repetition of sprint, the subject had to go back at the starting point by skating (mainly gliding) slowly. A 30 s passive recovery (including the slow skating time from the end point of one sprint trial to the beginning of the next) separated two consecutive sprints. Photocell automatic laser timing system (Mircrogate, Racetime 2, Bolzano, Italy) was used to measure sprint performance of the subject. Hand stopwatch was used to measure passive recovery period.

The subject performed two types of RSSA tests based on the durations of the passive recovery period:

RSSA-2: The passive recovery period between two consecutive sets was 2 min;

RSSA-3: The duration of the recovery period between two sets was 3 min.

##### Speed Decrement (S_dec_)

The speed decrement (%) in any given set of RSSA test was calculated by using the following formula [13]:

S_dec_ (%) = [(S1 + S2 + S3)/(3 × S_best_) − 1] × 100 (2)

where S1 to S3 are times of 3 sprint repetitions in a set, and S_best_ is the best sprint time in a set.

##### Warm-Up before RSSA Test

Duration and activities in warm-up in both RSSA-2 and RSSA-3 were similar. The subject warmed up for 20 min, which consisted of 15 and 5 min of off-ice and on-ice activities, respectively, spaced by about 20 min rest to put on hockey gear. The off-ice warm-up involved raising body temperature, mobilizing joints, activating muscles, and potentiating nervous system [19]. The on-ice warm-up comprised of alternating interplay of fast- and slow skating.

##### Recording of Heart Rate

Heart rate telemeter (Polar Team2, Polar Electro Oy, Kempele, Finland) was used to record heart rate, during SMAT, at an interval of 2 s. In RSSA tests, HR was recorded at an interval of 2 s, from warm-up till the end of the Set 6.

### 2.3. Statistical Analysis

To represent the average and the typical spread of values of all measurable variables, mean and standard deviation (SD) were used. Shapiro–Wilk test was used to verify the normal Gaussian distribution of the data. Levene’s and Mauchly’s tests were used to test homoscedasticity and sphericity of data, respectively. To determine differences between forwards and defensemen, Student’s *t*-test for independent samples was used for normally distributed data and equal variances, the t-Student test with Cochran-Cox adjustment in case of normally distributed data but unequal variances and the U-Mann–Whitney test for non-normally distributed data. The effect size (ES) of the intervention was calculated using Cohen’s guidelines. Threshold values for ES were >0.2 (small), >0.6 (moderate), >1.2 (large), and >2.0 (very large) [20]. To investigate differences in variables, a two-way analysis of variance with repeated measures and HSD (Honestly Significant Difference) Tukey-Kramer post hoc analysis was used. Statistical significance was set at *p* ≤ 0.05. All statistical analyses were conducted using Statistica 13.3 (TIBCO Software Inc., Palo Alto, CA, USA).

## 3. Results

### 3.1. Physical and Physiological Characteristics of the Subjects

Table 1 presents age, height, weight, muscle mass, body fat percentage, predicted VO_2max_, and HR_max_ of the subjects. Defensemen are taller (ES = Small), heavier (ES = moderate), have marginally higher body fat% (ES = Small) and muscle mass (ES = Moderate), and lower VO_2max_ (ES = small) than their forward counterparts.

### 3.2. RSSA-2- and RSSA-3 Test Performance

**Figure 2:**
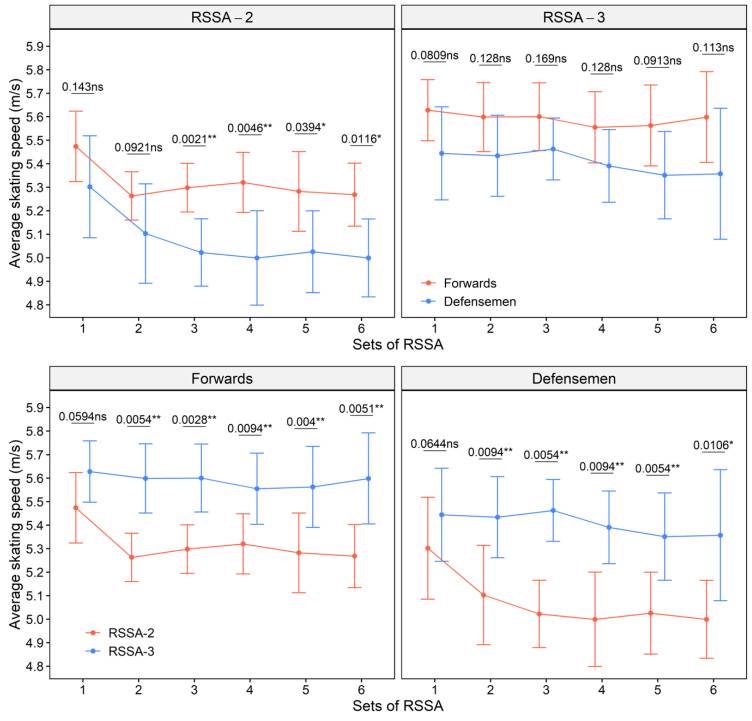
Average skating speed of the forwards and defensemen in RSSA-2 and RSSA-3 tests (ns—non-significant, * *p* < 0.05; ** *p* < 0.01).

Average skating speed of the forwards and defensemen in RSSA-2 and RSSA-3 tests is presented in Figure 2. Forwards performed both RSSA-2 and RSSA-3 faster than defensemen (the upper panel of Figure 2). The average skating speed of the forwards was significantly (*p* < 0.05) higher than the Defensemen in most of the sets of RSSA-2, except Sets 1 and 2. The difference of skating speed between forwards and defensemen; however, was not significant in RSSA-3. The lower panel of Figure 2 compares the skating performance of the subjects between RSSA-2 and RSSA-3 tests. Except in Set 1, subjects of both the playing positions skated much faster (*p* < 0.05) in RSSA-3 in comparison to RSSA-2.

### 3.3. Speed Decrement

**Figure 3:**
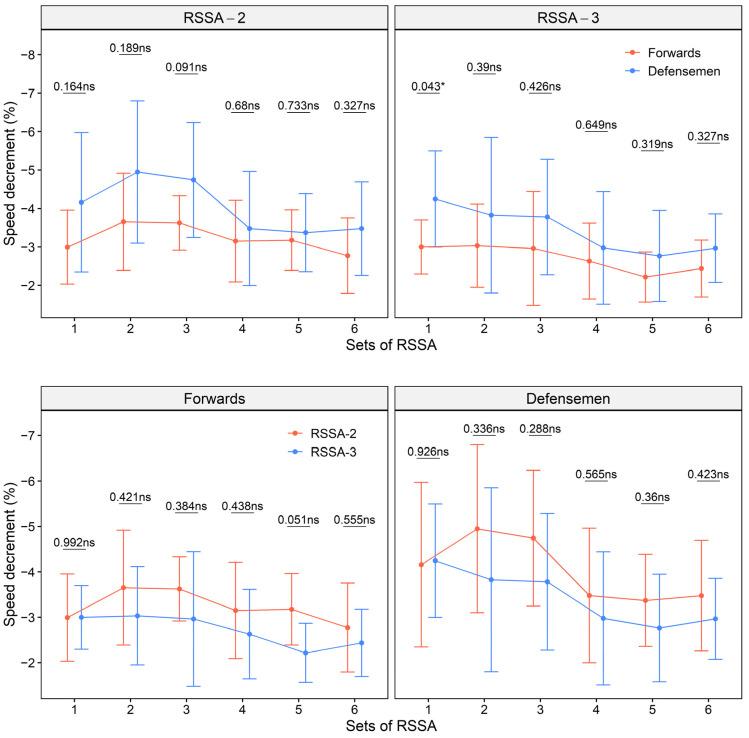
Speed decrement of the forwards and defensemen in RSSA-2 and RSSA-3 tests (ns—non-significant; * *p* < 0.05).

Figure 3 shows S_dec_ of the forwards and the defensemen in RSSA-2 and RSSA-3 tests. The S_dec_ is higher in RSSA-2 than RSSA-3, for any given set (except set 1), in both forwards and defensemen (the lower panel, Figure 3). The difference, however, is non-significant in all the cases. Defensemen showed higher S_dec_ than forwards in all the sets, although the difference was significant only in the Set 1 of RSSA-3 (the upper panel, Figure 3).

### 3.4. Heart Rate Response

**Figure 4:**
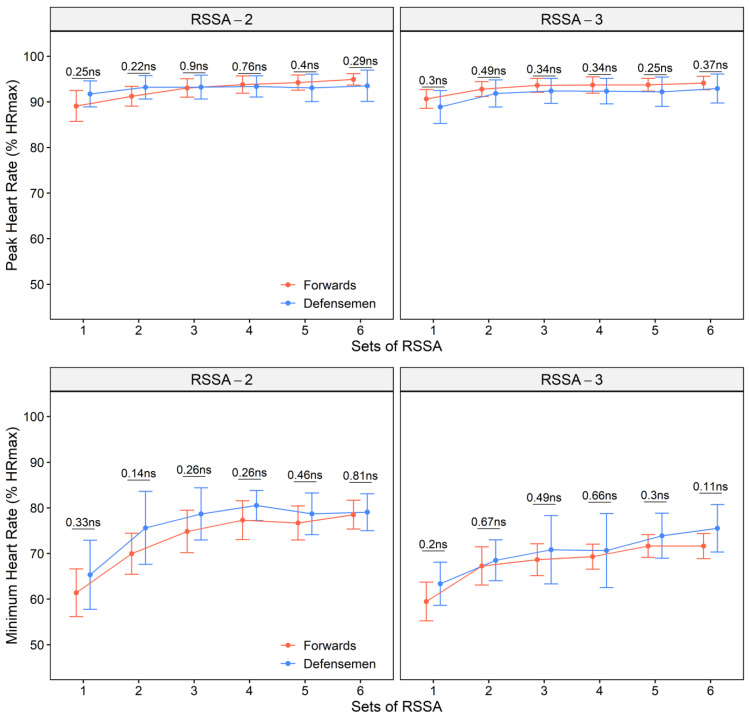
Peak HR and minimum HR of the forwards and defensemen during various sets of RSSA-2 (**above**) and RSSA-3 (**below**) tests (ns—non-significant).

Peak heart rate and HR_min_ attained in the subjects during RSSA-2 and RSSA-3 tests are presented in Figure 4. No difference in HR_peak_ was found between forwards and defensemen, and both attained highest HR_peak_ (forwards 94.9 ± 2.7 and 94.1 ± 2.2% HR_max_ in RSSA-2 and RSSA-3 respectively; defensemen 93.5 ± 3.5 and 92.9 ± 3.2% HR_max_ in RSSA-2 and RSSA-3, respectively) mostly in Set 6. Only a marginal rise in HR_peak_ was observed from Set 1 through 6. However, HR_min_ increased significantly higher from Set 1 through 6 in both forwards and defensemen.

## 4. Discussion

The major finding of this study is that the recovery period of 3 min (RSSA-3) between sets of repeated sprint causes both forwards and defensemen to skate faster and reduce S_dec_ when compared to a two minute recovery (RSSA-2). The forwards skate faster and show lower S_dec_ than defensemen, but HR response is similar in both forwards and defensemen.

The VO_2max_ of the participants of this study is like the top Polish ice hockey players [21], but inferior to ice hockey players of an elite category [1]. Lean body mass is one of the essential requirements for ice hockey players to support sprint and for optimizing performance, rapid change of direction, balance, agility, and frequent high impact body contact [10,22]. Higher division players were found to contain relatively lower body fat than lower division players of NCAA [23]. Twist et al. [10] reported that ice hockey goalkeepers contain higher body fat (13.5%) than defensemen (12.1%) and forwards (10.8%). The fat content of the subjects in this study is higher than elite ice hockey players, but similar to top Polish ice hockey players, as studied by Roczniok et al. [21]. Preseason measurement is one of the likely causes of higher body fat of the subjects in this study. Although excess fat and body mass are protective against collision during the game, these are major obstacles for skating due to increased frictional resistance on the ice [22]. Like many other studies, Defensemen in the present study also show higher body fat than forwards [22]. Higher physiological demand and training specificity are responsible for difference in body composition between forwards and defensemen [22].

Results show that a three minute passive recovery made a significant difference over a two minute recovery on average speed, but have moderate to low impact on S_dec_, and heart rate response in the subjects. An important fitness demand of an ice hockey player is the capability to recover quickly and to perform adequately in subsequent skating sprints [13]. For repeated sprint skating, phosphocreatine is the most important and immediate source of energy. The contribution of the oxidative phosphorylation is limited (<10%) during a single short sprint [24,25]. However, the magnitude of aerobic contribution increases progressively with repetitions of sprint and may contribute up to 40% of the total ATP generated in the final stage of a repetitive sprint exercise [24].

A large depletion of phosphocreatine occurs immediately after repeated sprint exercise, and longer than five minutes may be required for complete recovery of the phosphocreatine back to the pre-exercise level [26,27]. So, it is likely that only partial restoration of phosphocreatine takes place during two and three minute recovery period between the sets of RSSA. It is also expected that the recovery of phosphocreatine is more at the end of the three minute rest period than at the end of a two minute one. Gradual depletion of phosphocreatine at the beginning of subsequent sets of RSSA is likely to be an important factor that contributes to sprint decrement in the players.

Fatigue index (FI) and S_dec_ are two terms commonly used by researchers to quantify the capacity to resist fatigue and drop-off performance [16]. Speed decrement is possibly preferred over FI for the reason that FI is calculated from the drop-off in speed from the best to the worst, whereas S_dec_ considers all sprints [11,28]. So, S_dec_ may be lower in a set where all the repetitions are executed slowly. This may explain why the difference in S_dec_ between RSSA-2 and RSSA-3 was only marginal.

Peak HR in competitive ice hockey match play often exceeds 90% of HR_max_, whereas HR_aver_ varies around 85% of HR_max_ [22,29]. Gradual increase of HR with the progression of RSSA reflects the involvement of more energy from aerobic source during latter sets. The maximal cardiac workload in both the forms of RSSA is similar as indicated by similar HR_peak_, although the subjects experienced lower HR_min_ in RSSA-3. Shorter passive recovery in RSSA-2 is responsible for elevated HR before the next set begins. Relatively higher circulating plasma catecholamines before the onset of a set in RSSA-2 may be responsible for higher HR_min_ in RSSA-2 than in RSSA-3. Catecholamines, however, are short-lived signaling molecules in plasma [30], and their complete removal from circulation is unlikely to occur even three minutes after the end of a set.

The defensemen in this study were slower than forwards in both the formats and all the sets of RSSA test. Higher body weight, game demand, and training pattern of defensemen likely cause slower skating speed than forwards. Defensemen play longer than forwards in ice hockey match play due to higher number of shifts and thus shorter recovery period between the shifts [3,29]. Much slower skating speed also recorded in defensemen than Forwards during match play [3]. The game demand and difference in training may be other factors responsible for difference in average speed of the defensemen. However, despite faster repeated skating by the forwards, the HR_aver_ remains similar or marginally lower than defensemen. Superior endurance capacity of forwards may cause this result. Researchers reported very high HR_aver_ and HR_peak_ values both in forwards and defensemen during ice hockey match play, but show no significant difference between players of two different playing positions. Forwards and defensemen reported playing a match with HR_aver_ of 161 and 158 bpm, respectively, and attained HR_peak_ of 195.4 and 196.6 bpm, respectively [29,31]. In spite of slower movement than forwards, defensemen gave their maximum possible performance as indicated by HR_aver_.

### 4.1. Future Research Directions

To the best of our knowledge, studies on the effects of passive recovery period duration on the skating performance of ice hockey players are not known. Recovery period between two shifts of competitive ice hockey match play varies widely. This study can open a new avenue where the effects of various other recovery period durations (e.g., 2 versus 4 min, 3 versus 5 min, 3 versus 4 min) can be conducted. This can help coaches and players to find out the ideal bench time (or passive recovery time) in terms of maximum physiological benefits without affecting match strategies. A similar assessment of the sprint times and drop-off in repeated sprints efforts of the on-ice shift times would be informative for coaches. Moreover, similar studies can be conducted on ice hockey players of various levels.

### 4.2. Limitations

Low number of subjects, especially defensemen, is one of the major limitations of this study. Heart rate is the only physiological variable used in this study to analyze and compare stress in the two formats of repeated skating tests (i.e., RSSA-2 and RSSA-3). Observation and analysis of other variables like lactate, cortisol, and catecholamines can explain the metabolic basis of the findings of the study.

## 5. Conclusions

The results of the present study conclude (1) the benefits of a three-minute recovery period (RSSA-3), over two minutes (RSSA-2), in both forwards and defensemen by increasing the average skating speed, (2) forwards are faster and show slower drop-off performance than defensemen, and (3) HR response does not vary between forwards and defensemen during repeated sprint skating.

## Figures and Tables

**Table 1:** Physical characteristics of the volunteers.

Variables	Forwards (n = 12)	Defensemen (n = 7)	Δ (%)	*p*-Value	Effect Size
Age	23.4 ± 4.76	22.3 ± 5.2	1.2 (5.1%)	0.582	0.24/Small
Height(cm)	179.8 ± 5.68	182.0 ± 3.46	−2.2 (−1.2%)	0.331	0.43/Small
Weight(kg)	80.5 ± 7.57	87.1 ± 4.81	−6.6 (−8.2%)	0.036	0.97/Moderate
Body fat%	14.9 ± 4.75	17.3 ± 3.08	−2.4 (−16.1%)	0.208	0.56/Small
Muscle mass(kg)	39.2 ± 4.02	41.4 ± 2.19	−2.2 (−5.5%)	0.171	0.61/Moderate
Pred VO_2max_ (ml∙kg^−1^∙min^−1^)	52.3 ± 3.11	50.7 ± 6.24	1.6 (3.1%)	0.398	0.37/Small
HR_max_ (bpm)	197.8 ± 13.09	195.4 ± 5.78	----	----	----
